# Incorporation of zwitterionic materials into light-curable fluoride varnish for biofilm inhibition and caries prevention

**DOI:** 10.1038/s41598-019-56131-5

**Published:** 2019-12-20

**Authors:** Dohyun Kim, Myung-Jin Lee, Ji-Yeong Kim, Dasun Lee, Jae-Sung Kwon, Sung-Hwan Choi

**Affiliations:** 1 Department of Conservative Dentistry, Oral Science Research Center, Yonsei University College of Dentistry, Seoul, Republic of Korea; 2 Department and Research Institute of Dental Biomaterials and Bioengineering, Yonsei University College of Dentistry, Seoul, Republic of Korea; 3 Department of Orthodontics, Institute of Craniofacial Deformity, Yonsei University College of Dentistry, Seoul, Republic of Korea; 4 BK21 PLUS Project, Yonsei University College of Dentistry, Seoul, Republic of Korea

**Keywords:** Biomedical materials, Dental biomaterials, Fluoride varnish, Dental biofilms

## Abstract

We incorporated zwitterionic materials into light-curable fluoride varnish (LCFV) in order to inhibit biofilm accumulation and prevent dental caries, and the properties of LCFV with three different zwitterionic materials, namely, 2-methacryloyloxyethyl phosphorylcholine (MPC), carboxybetaine methacrylate (CBMA), and sulfobetaine methacrylate (SBMA) polymers (each at a weight percentage of 3%), were compared; unmodified LCFV without any zwitterionic material was used as a control. Material properties including film thickness and degree of conversion (DC) of each type of LCFV were evaluated. In addition, protein-repellent effects and inhibitory effects on *Streptococcus mutans* adhesion and saliva-derived biofilm accumulation of LCFV were estimated. Finally, the preventive effect of LCFV on enamel demineralization was assessed *in vitro* on extracted human teeth specimens stored in *S. mutans*-containing medium. The film thickness of LCFV significantly decreased with the incorporation of zwitterionic materials compared to the control LCFV, whereas there were no significant differences in the DC among all of the LCFV groups. Furthermore, the amount of adsorbed protein, adherent *S. mutans* colony-forming unit (CFU) counts, and saliva-derived biofilm thickness and biomass were all significantly lower for LCFV with incorporated zwitterionic materials compared with the control. All LCFV groups including the control showed certain preventive effects against enamel demineralization during a 14-day immersion in the medium with *S. mutans* and sucrose, and the depth of demineralization was significantly lower in LCFV with zwitterionic materials than in the control. Thus, the incorporation of zwitterionic materials such as MPC, CBMA, and SBMA appears to confer superior antifouling effects to LCFV.

## Introduction

While the prevalence of dental caries has decreased, it is still a significant oral health problem^1^, and cavity prevention is regarded as a priority for dental services as it is considered to be more cost-effective than treatment^2^. Topical fluoride is mainly used for prevention and noninvasive control of dental caries. It can be provided through various methods such as toothpaste, mouthrinse, gel, and varnish^3^. Fluoride varnish (FV) is a highly concentrated form of topical fluoride with a synthetic base, which is applied to the tooth surface for caries prevention, hypersensitivity treatment, or enamel remineralization. It was developed to prolong the contact time between the fluoride and tooth surface because the varnish adheres to the tooth surface for longer periods in a thin layer. Such FV has been used widely in dental clinics as a simple and effective way to protect the tooth surface against acid produced by cariogenic bacteria. Further, several reviews and meta-analyses have supported the effectiveness of FV in caries prevention^4–6^. However, because it is not a permanent varnish, repeated application is needed to maintain its effectiveness. It has been reported that the effectiveness of FV in reducing bacterial adhesion and biofilm accumulation decreased over time^7^.

Recently, light-curable fluoride varnish (LCFV) based on resin-modified glass ionomer (RMGI) has been introduced. It is considered to have advantages of longevity and sustainability, and it also provides good maneuverability with prolonged working times and short setting times achieved by light curing. It has been reported that the effectiveness of LCFV in the inhibition of enamel demineralization is comparable to conventional FV^8^. Moreover, recent clinical studies have shown that LCFV could effectively prevent enamel demineralization for a longer term compared with the conventional FV^9,10^. However, its surface is rougher than the conventional FV^11^, which results in more biofilm accumulation.

Zwitterionic materials are a family of materials that contain both cationic and anionic groups. They are characterized by high dipole moments and highly charged groups, and their overall charge is neutral^12^. The biomedical applications of zwitterionic materials have been investigated for a long time as they demonstrate antifouling properties owing to their unique molecular structures, and thus, the materials can function as resistant additives for protein adsorption and cell adhesion; they have been used for the surface treatment of artificial blood vessels, hip joints, microfluidic devices, etc.^13^. The three most widely studied molecules are phosphorylcholine (PC), carboxybetaine (CB), and sulfobetaine (SB), and the corresponding zwitterionic materials are 2-methacryloyloxyethyl phosphorylcholine (MPC), carboxybetaine methacrylate (CBMA), and sulfobetaine methacrylate (SBMA) polymers^14^.

MPC polymer is one of the most popular zwitterionic materials; it is a methacrylate with a phospholipid polar group in the side chain and has a strong protein-repellent property^15^. CB and SB are other water-soluble zwitterionic materials that belong to betaines, in which both cationic and anionic groups have the same monomer residue^16^. CB polymers have been used the longest among polybetaines and are regarded as very promising materials for a wide range of applications. SB polymers were first thought to be less antifouling than other polymers; however, they have been found to have ultralow fouling properties only with proper surface packing strategies^13^. MPC has been experimentally incorporated with dental materials such as resin-based composites, bonding agents, orthodontic cements, and calcium silicate cements to improve their antibacterial properties^17–20^. A recent study confirmed that LCFV containing MPC polymers showed noticeable antifouling effects on protein adsorption and bacterial adhesion, while maintaining original features such as physical properties and prevention of enamel demineralization^21^. However, thus far, there have been no reports on the other kinds of zwitterionic materials incorporated with dental materials.

Therefore, this study aimed to evaluate the incorporation of three different zwitterionic materials—MPC, CBMA, and SBMA polymers—into LCFV in regard to the inhibition of biofilm accumulation. Specifically, comparative analyses of the properties of LCFV with three zwitterionic materials incorporated and unmodified LCFV were carried out. The null hypothesis was that the incorporation of different zwitterionic materials would result in no significant differences in material properties, antifouling effects, and preventive effects on enamel demineralization of LCFV. The basic material properties such as film thickness and degree of conversion (DC) of LCFV were evaluated. In addition, the protein-repellent effects and inhibitory effects on *Streptococcus mutans* adhesion and saliva-derived biofilm accumulation of LCFV were estimated. Finally, the preventive effects of LCFV on enamel demineralization by *S. mutans* were assessed *in vitro* on extracted human teeth.

## Methods

### Incorporation of zwitterionic materials into LCFV

Three zwitterionic materials—MPC (Sigma-Aldrich, St. Louis, MO, USA), CBMA (TCI, Kita-ku, Tokyo, Japan), and SBMA (Sigma-Aldrich, St. Louis, MO, USA) polymers—were used in the study. Each zwitterionic material powder was mixed with LCFV (Clinpro XT Varnish; 3 M ESPE, St. Paul, MN, USA) at a weight percentage of 3%, which was found to be the most effective in a previous study^21^. All samples were prepared before light polymerization. The zwitterionic material powders were first mixed into a liquid-like state with the LCFV through hand-mixing. Then, the samples were vortexed followed by mixing using a high-speed mixer (SpeedMixer, Hauschild, Hamm, Germany), which has been shown to result in the thorough dispersion of powder^22^. The samples were polymerized by using a light curing unit (Elipar Free Light 2; 3 M ESPE, St. Paul, MN, USA) for 20 s according to the manufacturer’s instructions. The light intensity of the curing unit was 650 mW/cm^2^. Unmodified LCFV without any zwitterionic material was used as the control. Consequently, the following four groups were tested:

1. control LCFV (referred to as ‘control’)
2. 97.0% LCFV + 3% MPC (referred to as ‘LCFV + MPC’)
3. 97.0% LCFV + 3% CBMA (referred to as ‘LCFV + CBMA’)
4. 97.0% LCFV + 3% SBMA (referred to as ‘LCFV + SBMA’)

For the measurement of DC and tests for antifouling effects, disc-shaped specimens were prepared with 15-mm diameters and 2-mm thicknesses by using a custom-made mold. In each experiment, at least four specimens were tested for each group, and the experiment was repeated at least three times.

### Film thickness measurements

For the LCFV groups, film thickness was measured according to International Standard ISO 4049 with a digital micrometer (Mitutoyo Corporation, Tokyo, Japan) at a precision of 1 μm. First, the combined thickness of two stacked flat glass plates was measured. Subsequently, LCFV was placed between two flat glass plates and a load of 150 N was applied. After 180 s, the glass plates were removed from the loading device, and the thickness of the two plates was measured. The film thickness of LCFV was determined as the difference in thickness of the two glass plates before and after the placement of LCFV.

### Degree of conversion (DC)

The DC of each LCFV group was evaluated through Fourier transform infrared spectroscopy (FTIR) (Vertex 70; Bruker Optics, Ettlingen, Germany). The spectrometer was connected to a horizontal attenuated total reflectance (ATR) device consisting of a diamond crystal with a 2-mm diameter (Platinum ATR-QL; Bruker Optics, Baden-Württemberg, Germany). The diameter of the measured surface was 800 μm, the wave number range of the spectrum was 1400–2000 cm^−1^, and the FTIR spectra were recorded with two scans per second at a resolution of 4 cm^−1^. To determine the percentage of the remaining unreacted double bonds, the DC was assessed as the variation in the absorbance intensities peak area ratio of the methacrylate carbon double bond (peak at 1634 cm^−1^) and those of an internal standard (aromatic carbon double bond; peak at 1608 cm^−1^) during polymerization, in relation to the uncured material.

### Protein adsorption tests

The specimens were immersed in fresh phosphate buffered saline (PBS; Gibco, Grand Island, NY, USA) for 1 h at room temperature, followed by immersion into a protein solution of bovine serum albumin (BSA; Difco, Sparks, MD, USA) and bovine heart infusion (BHI; Difco, Sparks, MD, USA) broth (100 μL; 2 mg/mL) in PBS. After incubation at 37 °C for 1 h, the specimens were gently rinsed twice with fresh PBS. To assess initial protein adsorption on the specimen surface based on previous studies^23,24^, after 4 h of incubation under sterile humidity conditions at 37 °C in 5% CO_2_, any unadhered protein was removed by washing twice with PBS. The amount of adsorbed protein was measured by using 200-μL micro-bicinchoninic acid (Pierce Biotechnology Inc., IL, USA), followed by incubation at 37 °C for 30 min. The quantitative analysis of the adsorbed proteins on the LCFV specimen surface was performed by using the Micro BCA^TM^ Protein Assay Kit (Pierce Biotechnology, IL, USA). The optical density (OD) of each sample was measured by using a microplate reader (Epoch; BioTek Instruments, Winooski, VT, USA) at 562 nm.

### Bacterial adhesion analyses

*S. mutans* (ATCC 25175) cultured in BHI broth was used. The morphological changes of the bacteria on the surface of each LCFV specimen were examined by using a field emission scanning electron microscope (FE-SEM, Merlin; Carl Zeiss, Oberkochen, Germany). For sample preparation, 1 mL of the bacterial suspension (1 × 10^8^ cells/mL) was added to the LCFV specimens in a 24-well plate and the plates were incubated for 24 h. Then, the specimens were washed twice with PBS before fixing with 2% glutaraldehyde-paraformaldehyde in 0.1 M phosphate buffer (pH 7.4) for at least 30 min at room temperature. The specimens were then post-fixed with 1% OsO_4_ dissolved in 0.1-M PBS for 2 h, dehydrated in an ascending gradual series of ethanol, treated with isoamyl acetate, and subjected to a critical point dryer (EM CPD300; Leica, Wien, Austria). The specimens were coated with Pt (5 nm) by using an ion coater (EM ACE600; Leica, Wien, Austria) and examined by using the FE-SEM at 2 kV.

For the adhesion analysis, 1 mL of bacterial suspension (1 × 10^8^ cells/mL) was placed on the specimens and these were incubated at 37 °C for 24 h under aerobic conditions. After incubation, the specimens were gently washed twice with PBS to remove any non-adherent bacteria. The attached bacteria were harvested in 1-mL BHI by sonication in an ultrasonic cleaner (SH-2100; Saehan Ultrasonic, Seoul, Republic of Korea) for 5 min. Then, 100-µL harvested bacterial suspension was spread onto a solid agar plate and incubated for 24 h at 37 °C, and the total number of colonies was counted.

To confirm the viability of the adherent bacteria, the bacterial strain was stained by using a live/dead bacterial viability kit [SYTO9 and Propidium Iodide (PI); Molecular Probes, Eugene, OR, USA] according to the manufacturer’s instructions. PI staining is typically used for membrane integrity evaluations, however, when coupled with a universal stain such as SYTO9, staining of the nucleic acids can be performed irrespective of the membrane integrity. Moreover, when SYTO9 stains the nucleic acids of membrane compromised cells, on interaction with PI, SYTO9 is replaced with PI. Although this method has inherent limitations like other methods, it has been widely adopted in the literature for bacterial viability assessments^25,26^. Equal volumes of SYTO9 dye and PI were mixed thoroughly. From the mixture, 3 µL was added to each milliliter of the bacterial suspension. After 15 min of incubation at room temperature in the dark, the stained samples were observed by using a confocal laser microscope (LSM700; Carl Zeiss, Thornwood, NY, USA).

### Saliva-derived biofilm accumulation analyses

The protocol from previous studies was adopted, where human saliva was collected from healthy adult donors^22,27^. The saliva was obtained in accordance with the ethical principles of the 64th World Medical Association Declaration of Helsinki and following procedures approved by the institutional review board of the Yonsei University Dental Hospital (Seoul, Republic of Korea) (2-2019-0049). Written informed consent was obtained from all participants before donating saliva. The saliva donors were six adults without active dental caries or periodontal diseases, who had not consumed antibiotics within the past three months. The participants did not brush their teeth for 24 h and abstained from food/drink intake for at least 2 h prior to donating saliva. Their saliva was collected and mixed in equal proportions. The mixed saliva was then diluted in sterile glycerol to a concentration of 30% and stored at −80 °C to be used as a biofilm model. The biofilm model was cultured in McBain medium supplemented with mucin (type II, porcine, gastric) (2.5 g/L), bacteriological peptone (2.0 g/L), tryptone (2.0 g/L), yeast extract (1.0 g/L), NaCl (0.35 g/L), KCl (0.2 g/L), CaCl_2_ (0.2 g/L), cysteine hydrochloride (0.1 g/L), hemin (0.001 g/L), and vitamin K1 (0.0002 g/L) at 37 °C for 24 h. From the cultured medium, 1.5-mL bacterial solution was placed on the specimen. After 8, 16, and 24 h of incubation, an additional 1.5 mL of bacterial solution was placed on the specimen for each period, respectively. The biofilms were allowed to grow for a total of 48 h.

The specimens were stained with a live/dead bacterial viability kit (SYTO9 and PI) by using the same method described above for bacterial staining. Because the salivary-derived biofilm contains several species and the cultivability determined with colony-forming unit (CFU) data and viability data via fluorescent staining will differ in interpretations and are not correlated^28,29^, we used SYTO9 and PI in combination with a confocal laser microscope to facilitate approximately quantitative and spatial heterogeneity evaluations of the biofilm, thus providing an overview of the bacterial viability of the multi-species salivary microbiota^30^. The biofilm was visualized at five randomly chosen positions by using a confocal laser microscope (LSM700). The axially stacked biofilm images were captured, and the biofilm thickness was calculated by using the software (Zen; Carl Zeiss, Thornwood, NY, USA). In addition, the COMSTAT plug-in (Technical University of Denmark, Kongens Lyngby, Denmark) along with the ImageJ (NIH, Bathesda, MA, USA) software was used to determine the average biomass.

### Preventive effect on enamel demineralization

Randomly collected sound premolars from the human oral cavity obtained from the Yonsei University Dental Hospital Biobank resource base (Seoul, Republic of Korea) were used for the experiment following procedures approved by the institutional review board of the Yonsei University Dental Hospital (Seoul, Republic of Korea) (2-2019-0034). The teeth were cut in slices of 150-µm thickness by using a low-speed precision diamond cutter (DAIMO-100S; MTDI, Daejon, Republic of Korea) and 1200-grit SiC papers. A total of 60 specimens were obtained and randomly assigned into five groups, where a new group was added for no LCFV application. This negative control was specifically used for this experiment to verify that the medium containing *S. mutans* could demineralize tooth structures. For protection from indiscriminate surface demineralization, a bonding agent (Tetric N Bond Universal; Ivoclar Vivadent, Schaan, Lichtenstein) was applied on the sectioned surfaces of each specimen and light-cured for 20 s. A Mylar strip was placed over the surface before light curing to prevent the formation of an oxygen inhibition layer. Subsequently, nail varnish was applied around the circumference of each specimen, except on a 2 mm-enamel window, which was left exposed for demineralization. The enamel window was etched with 20% phosphoric acid for 30 s and rinsed with distilled water followed by the application of LCFV and light curing for 20 s, according to the manufacturer’s instructions. The specimens were placed in each well of a 12-well plate and exposed to BHI culture medium supplemented with 2% sucrose (1,492.5 μL/well) and 7.5 μL of *S. mutans* inoculum (1 × 10^8^ cells/mL) for 14 days. The growth medium was replaced every second day to support bacterial regrowth. After 14 days, the specimens were taken out and examined by using a microscope with a polarizing filter (BX41; Olympus, Tokyo, Japan) at 4X magnification.

### Statistical analyses

All statistical analyses were conducted by using SPSS 23 software program (IBM Corp., Armonk, NY). The results for different LCFV groups were presented as the median and interquartile range (IQR), and data were analyzed by Kruskal–Wallis tests with the Bonferroni correction. The level of significance was set as *P* < 0.05.

## Results

### Basic material properties

**Figure 1:**
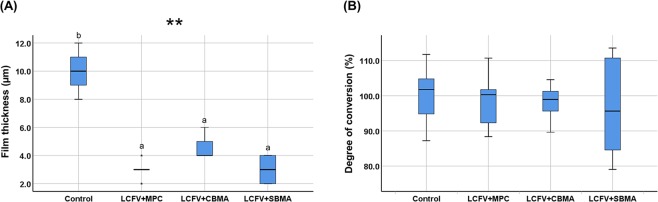
Basic material properties of LCFV containing different zwitterionic materials. (**A)** Film thickness. (**B**) Degree of conversion. Letters above the bars indicate significant differences (**P < 0.01).

The film thickness of the control (median, 10.0 μm; IQR, 3 μm) was significantly greater than those of LCFV with zwitterionic materials incorporated (*P* = 0.002, Fig. 1A). There were no significant differences in the DC among the different LCFV groups, thus indicating that the incorporation of zwitterionic materials did not affect the polymerization of LCFV (Fig. 1B).

### Protein adsorption

**Figure 2:**
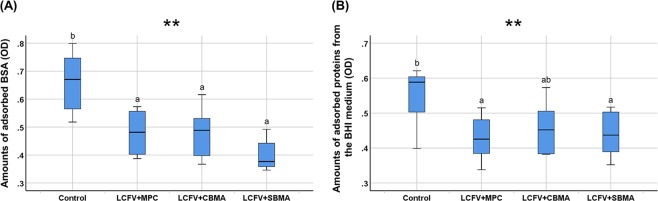
Protein-repellent effects of LCFV containing different zwitterionic materials. (**A**) Adsorbed bovine serum albumin (BSA). (**B**) Protein adsorbed from brain heart infusion (BHI) medium. Letters above the bars indicate significant differences (**P < 0.01).

The amount of absorbed BSA was significantly lower in the LCFV with zwitterionic materials incorporated than that in the control (OD value median, 0.67; IQR, 0.2) (*P* = 0.001), and there were no significant differences among the LCFV with different zwitterionic materials (Fig. 2A). The amount of adsorbed proteins from BHI medium was significantly lower for LCFV + MPC and LCFV + SBMA (OD value median, 0.42; IQR, 0.1 and OD value median, 0.43; IQR, 0.1, respectively) compared with the control (OD value median, 0.58; IQR, 0.1) (*P* = 0.003). There was no significant difference between LCFV + CBMA (OD value median, 0.45; IQR, 0.1) and the other LCFV groups (Fig. 2B).

### Bacterial adhesion

**Figure 3:**
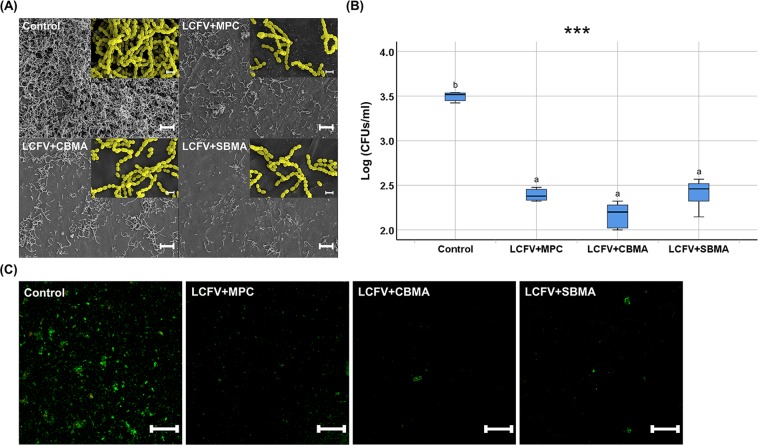
Bacterial adhesion (*S. mutans*) on the surfaces of LCFV containing different zwitterionic materials. (**A**) Scanning electron microscopy images (bottom, 1,000× ; top right, 10,000× ) of *S. mutans* cells attached on the surfaces of LCFV (scale bar: bottom, 10 µm; top right, 1 µm). To clearly distinguish between different magnification images, the color of the 10,000× image was changed to yellow by using software (3DSM Metrology Standard 7.3; Carl Zeiss, Thornwood, NY, USA). (**B**) Logarithmic colony-forming unit count values (CFUs/mL) derived from *S. mutans* cells attached on the surfaces of LCFV. Letters above the bars indicate significant differences (***P < 0.001). (**C**) Representative live/dead cell staining images of *S. mutans* cells attached on the surfaces of LCFV (scale bar = 500 µm).

Fewer *S. mutans* bacteria were observed on the surface of LCFV with zwitterionic materials incorporated compared to the control in the FE-SEM images (Fig. 3A). Further, in the quantitative analyses, the log (CFUs/mL) value (median, 3.51; IQR, 0.1) for the control was significantly higher than those for the other LCFV groups (*P* < 0.001); however, there were no significant differences in the CFU counts among the LCFV with three zwitterionic materials (Fig. 3B). These findings were confirmed by the cell viability staining results, where fewer live bacteria were found on the surfaces of LCFV with zwitterionic materials incorporated compared with the control (Fig. 3C).

### Saliva-derived biofilm accumulation

**Figure 4:**
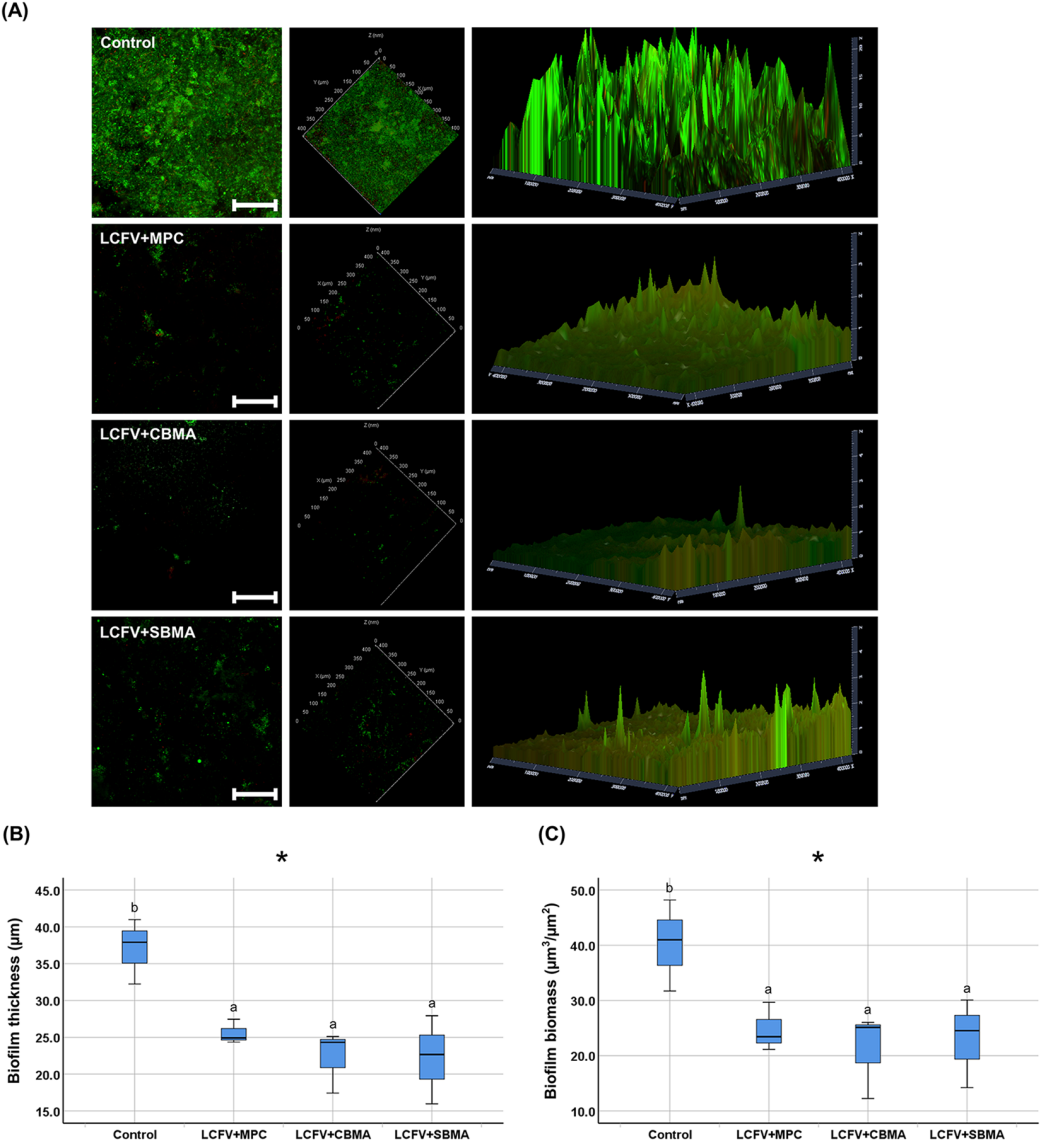
Saliva-derived biofilm accumulation on the surface of LCFV containing different zwitterionic materials. (**A**) Representative live/dead cell staining images of biofilms attached on the surfaces of LCFV (scale bar = 100 µm). Quantitative analysis of the (**B**) thickness and (**C**) biomass of the biofilms. Letters above the bars indicate significant differences (*P < 0.05).

The live/dead cell staining images were consistent with those obtained for *S. mutans* bacteria, which indicates that fewer multispecies bacteria adhered on the surfaces of LCFV with zwitterionic materials incorporated than the control (Fig. 4A). The incorporation of zwitterionic materials significantly decreased the thickness and biomass of the biofilm on the surfaces of LCFV (*P* < 0.05, Fig. 4B,C).

### Preventive effect on enamel demineralization

**Figure 5:**
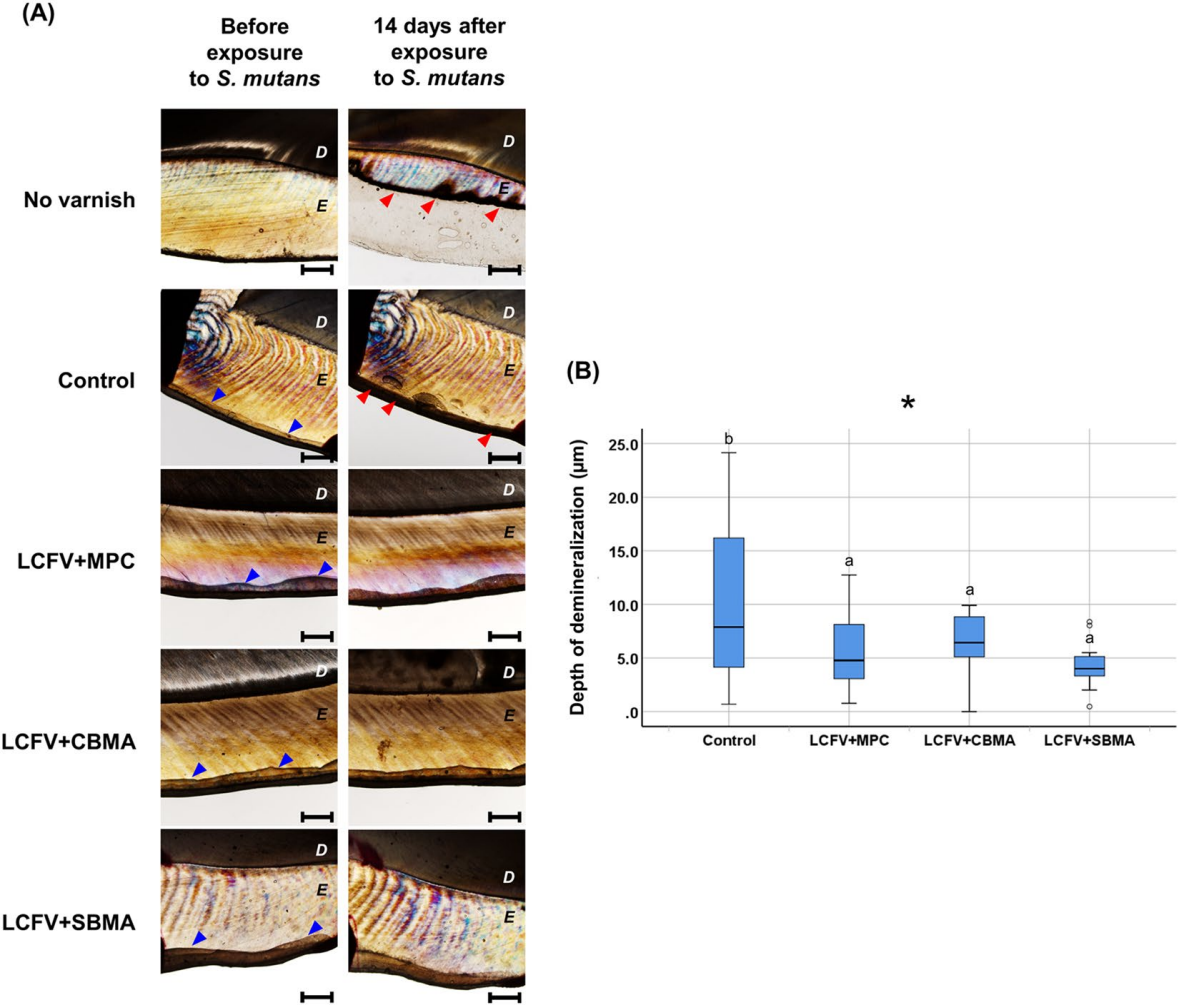
Demineralization of human enamel covered with LCFV containing different zwitterionic materials after exposure to BHI culture medium supplemented with 2% sucrose and *S. mutans* inoculum. (**A**) Representative polarized light microscopy images before and 14 days after exposure to *S. mutans* (scale bar = 500 µm, D: dentin, E: enamel). Blue arrowheads indicate LCFV covering enamel surfaces before exposure to *S. mutans*. Red arrowheads indicate the progression of dental caries 14 days after exposure to *S. mutans*. (**B**) Depth of demineralization 14 days after exposure to *S. mutans*. Different letters above the bars indicate significant differences (*P < 0.05).

After 14 days of immersion in the acidic medium resulting from the fermentation of sucrose by *S. mutans*, the specimens without the application of LCFV showed extensive demineralization and destruction of enamel layers (median, 366.4 μm; IQR, 95 μm), whereas all specimens with LCFV applied demonstrated preventive effects for enamel demineralization (Fig. 5A). The depth of enamel demineralization was significantly lower in the LCFV + SBMA (median, 4.01 μm; IQR, 2 μm), LCFV + MPC (median, 4.77 μm; IQR, 5 μm), and LCFV + CBMA (median, 6.43 μm; IQR, 4 μm) treatments than that in the control (median, 7.89 μm; IQR, 14 μm) (*P* < 0.05); there was no significant difference among the three LCFV groups with zwitterionic materials (Fig. 5B).

## Discussion

In the present study, three zwitterionic materials—MPC, CBMA, and SBMA polymers—were individually incorporated into the LCFV, and the properties of each group of LCFV as well as those of unmodified control LCFV were evaluated and compared. The null hypothesis was rejected as there were considerable differences in material properties, antifouling effects, and preventive effects on enamel demineralization between the LCFV with zwitterionic materials incorporated and the control. However, the properties of the LCFVs with three different zwitterionic materials were similar, thus suggesting that all three materials can be used with LCFV for biofilm inhibition.

There are several manners in which zwitterionic materials with biomaterials can be utilized, such as by incorporating the materials as surface coatings and grafting^14^. Surface coating or grafting strategies have mostly been used in medical devices that are grafted into the body or tissue. However, these surface modifications would not be suitable for dental materials, as they are exposed to an oral environment and the surfaces are susceptible to wear and loss. Therefore, the incorporation of zwitterionic materials would be more affordable and predictable for dental materials.

The concentration of zwitterionic materials was set at 3 wt% based on previous studies. There have been a few studies that investigated the optimal content ratios during incorporation of MPC polymers into dental materials, at which the materials could exhibit maximum antifouling effects without impairing the mechanical properties. Kwon *et al*.^21^ reported that with more than 5% of MPC incorporation, the film thickness of LCFV exceeded a value double that of the control; further, the protein-repellent and inhibitory effects for *S. mutans* adhesion were considerably reduced. They concluded that the best concentration to effectively inhibit bacterial adhesion while maintaining the original characteristics of the material itself was attained by 3% MPC incorporation into LCFV. Similarly, Zhang *et al*.^17^ reported that more than 3% of MPC incorporation considerably reduced the flexural strength and elastic modulus of resin-based composite, and at 3%, the best antifouling effect in terms of protein adsorption and bacterial adhesion on the composite surface was observed. The optimal proportion of CBMA and SBMA has not been reported yet. In our unpublished experiment, the physical properties of acrylic resin were decreased to below the ISO standard with more than 5% of CBMA incorporation. Fungal and bacterial adhesion and biofilm accumulation were the lowest at 3% of CBMA incorporation, and values were comparable to 3% MPC incorporation. Considering these results and equity among the different materials, the 3% concentration was selected in the present study.

The film thickness of LCFV significantly decreased when zwitterionic materials were incorporated, which is consistent with the results of a previous study^21^. The decreased film thickness would be more suitable for patients undergoing FV treatment. In contrast, it could affect the longevity or durability of the varnish as it eventually wears out or falls off the tooth. To this end, the LCFV was applied to a couple of layers before light curing. As the effect of the film thickness on the clinical performance of LCFV has not been explored yet, further investigations will be required.

During the progression of dental caries, saliva-derived proteins are adsorbed onto the tooth surface and then produce salivary pellicles, which serve as a medium for the attachment of bacteria^31^. Acidogenic bacteria ferment carbohydrates and produce organic acids, which diffuse into the tooth structures and dissolve mineral crystals^32^. *S. mutans* is well known to be important in the initiation of dental caries; further, bacterial biofilms are regarded as a key factor that directly contributes to the development of dental caries^33^. We used human saliva for the biofilm analyses because saliva has been regarded as an ideal material to cultivate biofilms *in vitro*, while maintaining the complex and heterogeneous natures of dental plaques *in vivo*^34^, which is advantageous. The results of this study confirmed a significant reduction in protein adsorption, *S. mutans* adhesion, and saliva-derived biofilm accumulation on the surfaces of the LCFV with zwitterionic materials incorporated compared with the control. The incorporation of each zwitterionic material into LCFV demonstrated an antifouling effect comparable to other materials. These *in vitro* experiments present certain limitations regarding the complicated environment in the oral cavity, such as salivary flow and various interactions between dental materials and surrounding tissues. Hence, further preclinical and clinical studies should be conducted to evaluate the clinical performances of the LCFV with zwitterionic materials incorporated.

Regarding the critical pH of 5.5 at which enamel demineralization is initiated^35^, it will be important for caries prevention to maintain higher pH values around the material. Zhang *et al*.^36^ reported that the pH of biofilm culture medium remained at about 6.5 for 48 h in the RMGI cement incorporated with 3% MPC even after water-aging for 30 days, whereas the pH decreased rapidly with time for all other RMGI cement groups. The authors stated that the antifouling property of MPC could repel bacteria and reduce acid production, thus resulting in a considerably higher pH. Even though our biofilm model was quite harsh, we could not measure the pH, and even though the enamel demineralization was more like an erosive lesion than a caries lesion in the present study, a similar scenario could be expected as the LCFV used in the study was a RMGI-based material. The protein-repellent effect and inhibition of biofilm accumulation on the LCFV with zwitterionic materials indicate that the material can act as a shield to enamel demineralization. Further investigations will be required to study the effects of zwitterionic material incorporation on pH change or fluoride release/uptake of RMGI-based dental materials.

There are some limitations in regard to the interpretation of the results from this study. Although additional protocols for washing adsorbents with solvents in a buffered solution prior to staining and testing in protein adsorption, bacterial adhesion, and biofilm accumulation can be used, we did not perform an elution step (e.g. analogous to ISO 7405 for biocompatibility testing for dental materials) before these experiments. Without elution before testing a potential pellicle-modifying or biofilm-repellant effect of a material, all findings may be limited to a short period of time after light-curing of the material (e.g. the first few hours) without any long-term effects when the first release-‘burst’ has abated.

Although zwitterionic materials certainly inhibited bacterial adhesion and biofilm accumulation in this study, bactericidal effects were not observed in the live/dead cell staining images. In fact, an antifouling effect does not equate to an antibacterial effect. There have been a few studies where the addition of quaternary ammonium methacrylates (QAMs) could help to establish antibacterial properties in dental materials. Wang *et al*.^37^ reported that the resin-based composite containing of 3 wt% MPC and 3 wt% dimethylaminododecyl methacrylate (DMAHDM) could suppress not only cariogenic bacteria but also periodontal pathogens. Further experiments are required to evaluate the effect of incorporating similar other antibacterial agents with the LCFV. In the same context, as CBMA or SBMA polymers have not been incorporated into dental materials since the present study, further research regarding the incorporation of other various zwitterionic materials into dental materials would be valuable.

In this study, human teeth specimens were used to evaluate the caries preventive effect of control and experimental LCFVs. To induce dental caries, the specimens were immersed in a BHI medium containing 2% sucrose and *S. mutans*, and polarized microscopy was used to measure the demineralization area^21^. Polarized light microscopy has been widely used to evaluate and measure the demineralization area of teeth specimens, especially for the evaluation of demineralization inhibition or remineralization effects of various materials or agents^38–40^. The extensive demineralization was shown in the negative control, which did not receive a LCFV application, indicated that the medium could effectively induce dental caries in the enamel surface (Fig. 5A). Among the LCFV groups, the depth of enamel demineralization was significantly lower in the LCFV with zwitterionic materials compared to the control, thus demonstrating that the LCFV with zwitterionic materials incorporating had a superior caries inhibitory effect than the control LCFV in human teeth specimens under the designed experimental circumstances. The improved antifouling effect and preventative effect on caries achieved by incorporating zwitterionic materials might have been related to the interference in the attachment of bacteria on the tooth surface. Further studies on the durability and longevity of these experimental LCFVs on the tooth surface are needed. Furthermore, clinical research on the effectiveness of LCFV containing zwitterionic materials should be conducted to provide further information and the development of appropriate clinical recommendations.

Within the limitations of the present study, the results clearly indicate that the incorporation of zwitterionic materials, such as MPC, CBMA, and SBMA, can provide superior antifouling effects to LCFV, without negatively influencing the original material properties. These zwitterionic materials can improve not only the inhibitory effect of LCFV against bacterial adhesion and biofilm accumulation, but also the preventive effect toward dental caries. This study considers just one aspect of the beneficial relationship between zwitterionic materials and dental materials. Considering that the antifouling effect of zwitterionic materials is actively used in various biomedical studies, it could certainly contribute to the advancement of many dental materials, as well as prove to be beneficial for clinicians and patients.
